# Identification of missense variants in the C-domains of von Willebrand factor that cause gain-of-function–like activity

**DOI:** 10.1182/bloodadvances.2025018101

**Published:** 2026-03-06

**Authors:** Golzar Mobayen, Yi Liu, Fan Gong, Omid Seidizadeh, Timea Feller, Alain Chion, Ana Ignat, Michael Appiah, Stephen Rothery, X. Frank Zhang, Thomas A. J. McKinnon

**Affiliations:** 1 Department of Immunology and Inflammation, Centre for Haematology, Imperial College, London, United Kingdom; 2 Department of Biomedical Engineering, University of Massachusetts Amherst, Amherst, MA; 3 Fondazione IRCCS Ca’Granda Ospedale Maggiore Policlinico, Angelo Bianchi Bonomi Hemophilia and Thrombosis Center, Milan, Italy; 4 Discovery and Translational Science Department, Leeds Institute of Cardiovascular and Metabolic Medicine, University of Leeds, Leeds, United Kingdom; 5 Molecular and Nanoscale Physics Group, School of Physics, University of Leeds, Leeds, United Kingdom; 6 Irish Centre for Vascular Biology, School of Pharmacy and Biomolecular Sciences, Royal College of Surgeons in Ireland, Dublin, Ireland; 7 National Heart and Lung Institute, Imperial College, London, United Kingdom

## Abstract

Recently characterized mutations Phe2561Tyr, Pro2555Arg in the von Willebrand factor (VWF) C4 domain, and Gly2705Arg in the C6 domain reportedly confer gain-of-function–like activity on the molecule, increasing responsiveness to shear stress, resulting in enhanced platelet capture. This implies that the C-terminal stem, comprised of the D4-C6 domains, plays a crucial role in modulating VWF function. To further investigate this, we used site-directed mutagenesis to generate a panel of uncharacterized C-domain variants. VWF expression and function were analyzed under static and shear stress conditions, and the biophysical properties of the variants were characterized by single-molecule optical tweezers (OT), and atomic force microscopy (AFM) imaging. All the expressed variants exhibited normal function in static assays, except the C1 domain Arg2287Trp variant that demonstrated increased binding to GPIbα. Under flow conditions at 1500 per second, all the variants had normal VWF-mediated platelet capture to collagen; however, at 5000 per second Arg2287Trp demonstrated enhanced platelet capture, whereas Asn2636Tyr (C5 domain), Thr2647Met (C6 domain), and Gly2705Arg showed reduced activity. Further analysis of the formation of rolling VWF-platelet aggregates over a VWF surface demonstrated an enhanced response to shear stress for the Arg2287Trp and Arg2384Trp (C2 domain) variants. OT analysis identified novel extension events exclusive to the C-terminal domains and more frequent unfolding events and longer unfolding extensions for Arg2287Trp and Arg2384Trp, consistent with increased flexibility and stem opening. AFM imaging confirmed that both variants favored the open stem conformation. Together, we demonstrate that mutations in the C1 and C2 domains alter stem dynamics, rendering VWF more responsive to shear stress.

## Key Points

• The C-domains of VWF form a stem structure that modulates response to shear stress.
• Arg2287Trp and Arg2384Trp are 2 novel variants that alter stem dynamics, leading to gain-of-function–like activity.

## Visual Abstract

**Figure:**
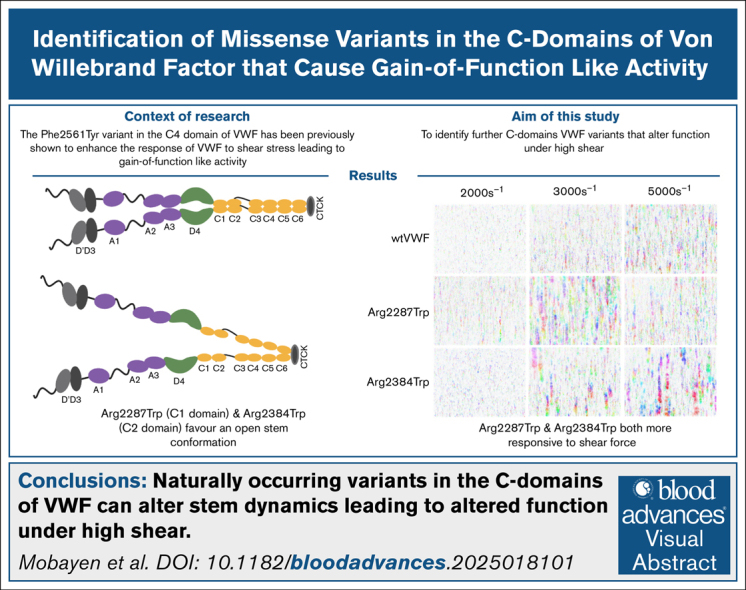

## Introduction

Von Willebrand factor (VWF) is a large multimeric plasma glycoprotein that plays a crucial role in hemostasis by acting as the carrier molecule for factor VIII and supporting platelet adhesion under shear stress.^1^^,^^2^ Central to its functional role is the large multimeric size of VWF, with the largest multimers being the most hemostatically active.^3^ Deficiency of VWF or perturbed VWF functions can lead to the bleeding disorder von Willebrand disease (VWD), which varies in severity from asymptomatic to life-threatening bleeding episodes.^4^^,^^5^ In contrast, a failure to process ultralarge multimers by the enzyme ADAMTS13 results in thrombotic thrombocytopenic purpura, characterized by widespread microvascular occlusions.^6^^,^^7^ Moreover, VWF has been implicated in thrombotic-associated disease, with *Vwf*^−/−^ mice demonstrating reduced atherosclerotic plaque formation and protection against stroke and deep vein thrombosis.^8^, ^9^, ^10^, ^11^ In humans, elevated plasma VWF levels have been associated with an increased risk of cardiovascular-associated diseases.^12^, ^13^, ^14^, ^15^, ^16^, ^17^, ^18^, ^19^, ^20^, ^21^, ^22^ For example, increased levels of VWF are associated with increased risk of myocardial infarction and ischemic stroke.^23^

Multimers are comprised of a series of monomers linked together by N- and C-terminal disulfide bonds, with each monomer containing a set of repeating domains arranged as: D1D2-D′D3A1A2A3D4C1C2C3C4C5C6CK.^24^ The C-terminal region of the molecule, taken as the D4 assembly to the CK domain, has been previously shown to form a pH-dependent dimeric stem structure that aids packaging into Weibel-Palade bodies following synthesis in endothelial cells.^25^ The exact factors modulating stem formation are not yet fully understood; however, the D4 assembly appears to be critical for proper stem formation and may be aided by Ca^2+^ ions.^26^ Moreover, although it was initially assumed that the stem structure was relaxed following secretion from Weibel-Palade bodies into the more neutral pH of the circulation, recent data suggests that the stem can persist in both open and closed forms.^27^ As well as the D4 assembly, work from Lof et al^28^ proposed that the C-domains, which form most of the stem region, can “unzip” in response to force, and this is likely to be important for VWF function under shear stress.

Although many putative VWD-causing mutations have been characterized, the functional effects of missense substitutions in the VWF C-domains remain poorly investigated. These domains harbor numerous reported variants with unknown consequences, although we recently described 9 previously uncharacterized type 1 and type 3 VWD variants in the C-terminal portion of VWF.^29^ Interestingly, the common p.Tyr2561Phe variant located in the C4 domain has been previously linked to an increased risk of cardiovascular disease rather than bleeding, with Phe2561 shown to be an independent risk factor for multiple myocardial infarction events in women under the age of 55.^30^ Functional analysis of this variant demonstrated that under shear stress using plate and cone aggregometry, Phe2561 carriers formed larger platelet aggregates, and recombinant Phe2561 had a reduced shear stress threshold to form rolling VWF-platelet aggregates compared to Tyr2561.^30^ It was hypothesized that Tyr2561 perturbed the conformation of the stem structure, resulting in a more open molecule with an enhanced response to shear forces, demonstrating gain-of-function (GoF)–like activity. Subsequently, 2 other putative GoF mutants have been identified: p.Pro2555Arg in the C4 domain and p.Gly2705Arg in the C6 domain.^31^^,^^32^ This raises the possibility that other mutations may exist within the C-domains that confer GoF-like activity to VWF rather than loss of function.

In the present study, we have selected a panel of reported variants in the C-domains of VWF that are not directly associated with VWD or where the mutation may have an effect, but no experimental proof exists. Strikingly, we identified 2 variants, p.Arg2287Trp and p.Arg2384Trp, located in the VWF C1 and C2 domains, respectively, that exhibited reduced expression but demonstrated GoF-like activity under shear conditions. Our novel findings indicate that the C1 and C2 domains of VWF may play a crucial role in mediating VWF function under vascular shear stress.

## Methods

### VWF variant selection

VWF genetic variants were selected from the Genome Aggregation Database (gnomAD), version 4.1, which contains genetic data from 807 162 individuals, by performing a search for “*VWF*” and selecting “missense/inframe indel” variants only. Known pathogenic variants were excluded, with an emphasis on benign or variants of unknown significance. Literature searches were performed on selected variants using PubMed and Google with the search terms “VWD or VWF” and the name of the variant in either single letter or triplet amino acid code, that is p.P2561Y or p.Phe2561Tyr. The variant Asp2636Tyr was found during online searches but was not in the gnomAD database and was included in this study. Accordingly, 11 final variants were selected for our analyses, including the previously characterized Phe2561Tyr. Combined Annotation Dependent Depletion analysis, as a computational in silico tool, was used to assess the potential functional impact of these genetic variants.^33^ The minor allele frequency of these variants across diverse populations was obtained from gnomAD, version 4.1.

### VWF variant generation, expression, and static binding analysis

Variants were introduced into VWF cDNA using site-directed mutagenesis and were expressed in HEK293T cells as previously described.^34^ Analysis of VWF expression and multimer composition, collagen binding, and binding to glycoprotein Ib and glycoprotein IIbIIIa was performed as previously described.^34^, ^35^, ^36^ Detailed methodology can be found in the supplemental Methods.

### VWF-mediated platelet capture to collagen

Ibidi VI^0.1^ flow slides were coated with 100 μg/mL human type III collagen (Southern Biosciences) overnight at room temperature in a humidified bag. Channels were washed 3 times with sterile phosphate-buffered saline (PBS) and blocked with 2% bovine serum albumin in PBS for 60 minutes prior to perfusion. Whole blood was freshly drawn from healthy volunteers not on any medication and was anticoagulated with 10% v/v acid citrate dextrose. Washed red blood cells and platelets (plasma-free blood) were prepared as previously described, and platelets were labelled with DiOC_6_.^36^^,^^37^ Plasma-free blood supplemented with 2.5 μg/mL wild type (wt) or variant VWF was perfused over the collagen surface at 1500 per second or 5000 per second for 5 minutes, and platelet capture was recorded in real-time, and platelet surface coverage was determined using ImageJ software.

#### Formation of rolling VWF-platelet aggregates

Ibidi VI^0.1^ flow slides were coated with 30 μg/mL wtVWF diluted in PBS for 2 hours at room temperature and following washing 3 times with PBS were blocked with 2% bovine serum albumin in PBS for 60 minutes prior to perfusion. Plasma-free blood was supplemented with 5 μg/mL wt or variant VWF and perfused through the channels for 60 seconds at 1000 per second, then the shear rate increased to 2000 per second for 60 seconds, then increased to 3000 per second for 60 seconds, then finally 5000 per second for 60 seconds. The final 15 seconds of each shear rate was recorded in real-time at 21 frames per second. The formation of rolling aggregates was assessed using an ImageJ macro. This macro creates a smoothed and filtered mask image of the stationary background objects, which is then subtracted from the original data series, resulting in an image series with only the dynamic objects enhanced for detection. The series is then color-coded against time and projected to display the result. Aggregate size was determined using ImageJ and expressed as arbitrary units.

### Optical tweezer pulling analysis

A dimeric VWF molecule spanning the D′-CK domains was expressed with N-terminal Spy or Avi-tags, the latter allowing for site-specific biotinylation. The biotinylated Avi-Tag allowed VWF to be captured on a fixed streptavidin-coated bead. Subsequently, the spy-tag was captured by the spy-catcher protein that was coupled to a DNA handle linked to a laser-captured bead as previously described.^38^ Through this interaction, a single VWF dimer was pulled by applying force (0-100 pN) through an approach and retract mechanism at various pulling speeds (50, 100, 200, 400, 500 nM/s) and the distribution of unfolding events was determined.

### AFM

Dimeric VWF D′-CK proteins (5 μg/mL) were applied to mica surfaces and allowed to coat for 30 seconds before being washed with high-purity H_2_O and then allowed to dry. Samples were imaged in 1 μm^2^ sections using a JPK NanoWizard atomic force microscopy (AFM) using an SNL-A probe. Analysis of the conformation of the stem state was performed as previously described.^27^

### Data analysis

Data analysis was performed using Prism Software for Science software package (version 10.0; GraphPad Software). Results are presented as standard deviation (SD) with means. The statistical significance of differences between groups was assessed using 1-way analysis of variance with Sidak’s multiple comparisons.

## Results

### VWF variant selection

**Figure 1.**
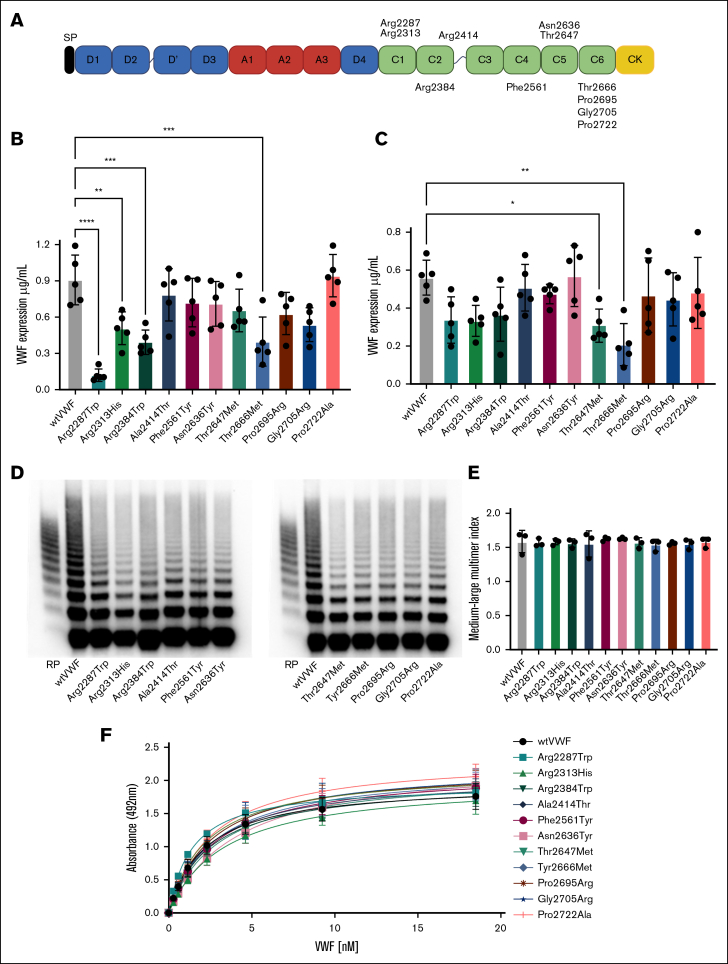
**Expression, multimeric structure, and collagen binding function of recombinant VWF C-domain variants.** HEK293T cells were transfected with full-length VWF expression vectors for the C-domain variants. Conditioned media and lysates were harvested 72 hours posttransfection, and the concentration of VWF was quantified using ELISA. (A) Schematic of the VWF monomer structure, with the domain location of the variants analyzed in this study. (B) Media VWF levels were significantly reduced for the Arg2287Trp, Arg2313His, Arg2384Trp, and Tyr2666Met variants. (C) Lysate VWF levels were slightly but significantly reduced for the Thr2647Met and Tyr2666Met variants. (D) Media from large-scale expression in HEK293T cells was concentrated, and the multimeric pattern was assessed in 1.2% multimer gels. All the mutants demonstrated a similar multimeric profile compared to wild-type VWF. (E) Concentrated media from 3 separate transfections were assessed by multimer analysis and multimer gels scanned using densitometry. The medium-large multimer index was determined and showed no differences between wtVWF and variant VWF. (F) Collagen binding was determined using a static collagen binding assay with plates coated with human type III collagen. Similar binding affinity was observed for all recombinant proteins. Error bars represent the mean SD of 3 to 5 biological repeats performed in duplicate. ∗*P* < .01; ∗∗*P* < .001; ∗∗∗*P* < .0001; ∗∗∗∗*P* < .00001. ELISA, enzyme-linked immunosorbent assay; SP, signal peptide; CK, cysteine knot.

Eleven *VWF* variants were selected from the gnomAD version 4.1.0 database and/or literature: p.Arg2287Trp, p.Arg2313His, p.Arg2384Trp, p.Ala2414Thr, p.Phe2561Tyr, p.Asn2636Tyr, p.Thr2647Met, p.Tyr2666Met, p.Pro2695Arg, p.Gly2705Arg, and p.Pro2722Ala. Table 1 reports the global minor allele frequency of the variants, their distribution across diverse gnomAD populations, and the corresponding Combined Annotation Dependent Depletion scores. All variants were rare (minor allele frequency [MAF] <1%), except p.Phe2561Tyr (MAF 4.3%) and p.Gly2705Arg (MAF 5.8%), which were common. Figure 1A shows the domain location of the 11 variants.

**Table 1.** **Frequency of VWF C-domain variants**

Genetic ancestry group	p.Arg2287Trp	p.Arg2313His	p.Arg2384Trp	p.Ala2414Thr	p.Phe2561Tyr	p.Asn2636Tyr	p.Thr2647Met	p.Thr2666Met	p.Pro2695Arg	p.Gly2705Arg	p.Pro2722Ala
African/African American	0.008928	0.002385	0.002223	NF	0.02249	NF	0.0006531	0.06984	0.00005332	0.01697	0.00001335
Admixed American	0.0005498	0.0004998	0.0009668	0.00003335	0.02860	NF	0.0005498	0.005267	0.0002665	0.05973	0.00001667
Ashkenazi Jewish	NF	NF	NF	NF	0.02753	NF	0.001723	0.0009125	NF	0.03358	NF
East Asian	NF	NF	NF	0.00002230	0.00002228	NF	0.00006686	NF	NF	0.002117	0.00004456
Middle Eastern	0.001980	0.0001650	NF	0.0001645	0.02062	NF	0.0003299	0.002310	NF	0.05856	NF
European (non-Finnish)	0.00001610	0.0002314	0.00005617	0.000003390	0.04891	NF	0.004119	0.0001517	0.0005737	0.05767	0.0001322
European (Finnish)	NF	NF	NF	0.00001565	0.07710	NF	0.01916	0.00003125	0.00001562	0.05946	NF
South Asian	0.00003294	0.00006587	0.00001105	0.00002196	0.004040	NF	0.0002196	0.0001867	NF	0.1247	NF
Remaining	0.0005119	0.0004479	0.0003141	NF	0.03747	NF	0.003568	0.005089	0.0006079	0.05754	0.00003201
XX	0.0005243	0.0003163	0.0002215	0.000007388	0.04387	-	0.004064	0.004144	0.0004899	0.05591	0.0001022
XY	0.0004279	0.0003243	0.0001660	0.000006240	0.04255	-	0.003949	0.003424	0.0004216	0.05958	0.00009860
Total MAF	0.0004764	0.0003203	0.0001940	0.000006818	0.04321	-	0.004007	0.003787	0.0004560	0.05773	0.0001004
CADD score	24	7	28	0.0360	17.7	5.9	3.8	9	24.4	29.0	15.3
ClinVar	VUS	VUS	VUS	VUS	Benign	Likely benign	VUS	Likely benign	VUS	Benign	VUS

General guidelines for interpretation of CADD score are as follows:

CADD PHRED <10: likely benign; similar to common neutral variants.

CADD PHRED, 10-20: moderately deleterious; may have some functional impact but not necessarily pathogenic.

CADD PHRED >20: likely pathogenic; among the top 1% most deleterious variants.

CADD PHRED >30: highly deleterious; among the top 0.1% most damaging variants in the genome.

CADD, Combined Annotation Dependent Depletion; MAF, minor allele frequency; NF, not found; VUS, variant of uncertain significance.

### Expression of VWF C-domain variants

**Figure 2.**
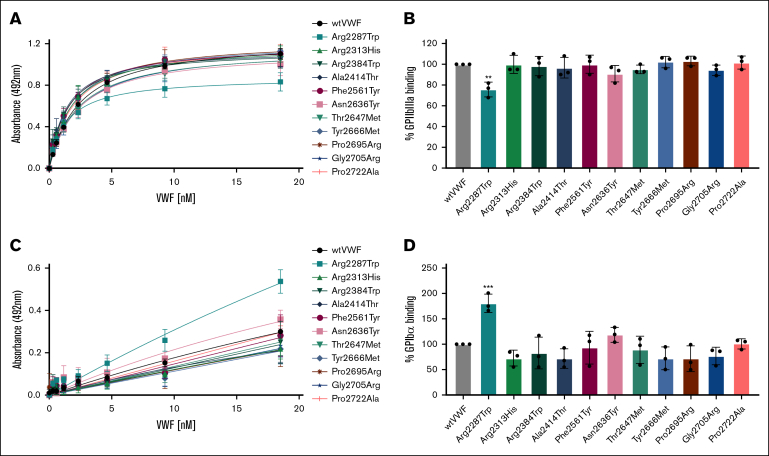
**Binding of VWF C-domain variants to GPIIbIIIa and GPIbα.** Microtiter plates coated with platelet-derived GPIIbIIIa were incubated with the recombinant VWF variants, and the bound protein was detected with anti-VWF-HRP antibodies. (A) Binding isotherms and (B) end point analysis showed no significant difference in GPIIbIIIa binding for the C-domain variants, with the exception of a modest, but significant reduction in binding for the Arg2287Trp variant upon end point analysis. Binding to GoF-GPIbα was assessed in the absence of ristocetin. (C) Binding isotherms failed to plateau for all the proteins, but a clear increase in binding was seen with Arg2287Trp. (D) End point analysis confirmed significantly increased binding for Arg2287Trp compared to wild-type VWF. Error bars represent the mean SD of 3 biological repeats performed in duplicate. ∗∗*P* < .001; ∗∗∗*P* < .0001.

To determine the effect of the C-domain variant panel on expression, we transfected HEK293T cells and analyzed VWF levels in the secreted media and cell lysates. Significantly reduced secretion was observed with the p.Arg2287Trp variant to ∼25% of wtVWF (Figure 1A). Moreover, a relatively small but significant reduction in secretion was also seen with the p.Arg2313His, p.Arg2384Trp, and Tyr2666Met variants, indicating these may alter proper VWF expression and could potentially be associated with VWD or borderline VWF levels (Figure 1A). VWF levels in the cell lysates were all broadly comparable to wtVWF, although significantly lower lysate VWF levels were observed with the Thr2467Met and Tyr2666Met variants (Figure 1B). Cotransfections performed with an expression vector encoding green fluroescent protein showed similar levels of green fluroescent protein expression, indicating that differences in expression are attributed to the VWF variant being expressed and not due to differences in transfection efficiency (supplemental Methods; Figure 2). Following large-scale expression of the proteins, we assessed their multimeric pattern and collagen-binding function. All the variants formed a full range of multimers like wtVWF (Figure 1C). Multimer bands were analyzed using densitometry, and multimer distribution quantified using the medium-large multimer index,^39^ with no differences in the multimer index between wtVWF and the variants (Figure 1D). This was supported by static collagen binding assays (Figure 1E) with all the variants exhibiting similar binding isotherms to wtVWF, and demonstrating similar K_D,app_ values all within the normal expected range of 1 to 10nM.^40^

### Binding of VWF C-domain variants to platelet receptors

We next investigated if any of the variants affected binding to GPIIbIIIa under static conditions. Except for p.Arg2287Trp, all variants bound GPIIbIIIa with similar affinity as wtVWF (Figure 2A). Interestingly, although p.Arg2287Trp bound GPIIbIIIa with similar affinity as wtVWF; it showed slightly reduced binding on end point analysis, highlighting a small, but statistically significant reduction in binding (Figure 2B). Next, we assessed binding of the variants to a GoF-GPIbα variant under static conditions. Interestingly, although most variants showed broadly similar binding profiles to GoF-GPIbα (Figure 2C), the p.Arg2287Trp variant exhibited apparent enhanced binding, which was statistically significant on end point analysis (Figure 2D). Together, these data demonstrates that p.Arg2287Trp has altered binding properties to platelet receptors, at least under static conditions.

### VWF-mediated platelet capture to collagen under shear stress

**Figure 3.**
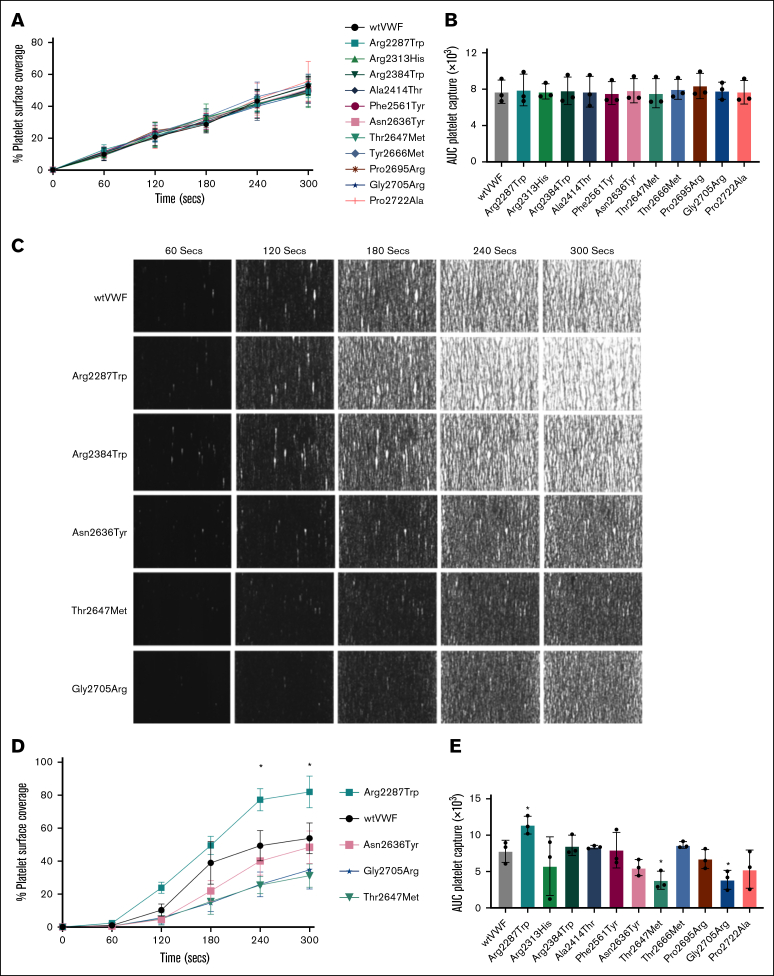
**VWF-mediated platelet capture to collagen under flow.** Ibidi VI^0.1^ flow slides coated with human type III collagen were perfused with plasma-free blood supplemented with 5 μg/mL VWF at 1500 per second, and platelet capture was recorded in real-time. (A) Platelet capture plotted over time, and (B) AUC analysis showed no significant differences in function. (C) Flow assays were repeated at 5000 per second. Representative time course images for the Arg2287Trp, Asn2636Tyr, Thr2647Met, and Pro2695Arg variants. (D) Analysis of surface coverage over the time course showed significantly increased platelet capture for Arg2287Trp at 4 minutes and 5 minutes. (E) AUC analysis for all mutants at 5000 per second. Error bars represent the mean SD of 3 biological repeats performed in duplicate. ∗*P* < .01. AUC, area under the curve; secs, seconds.

**Figure 4.**
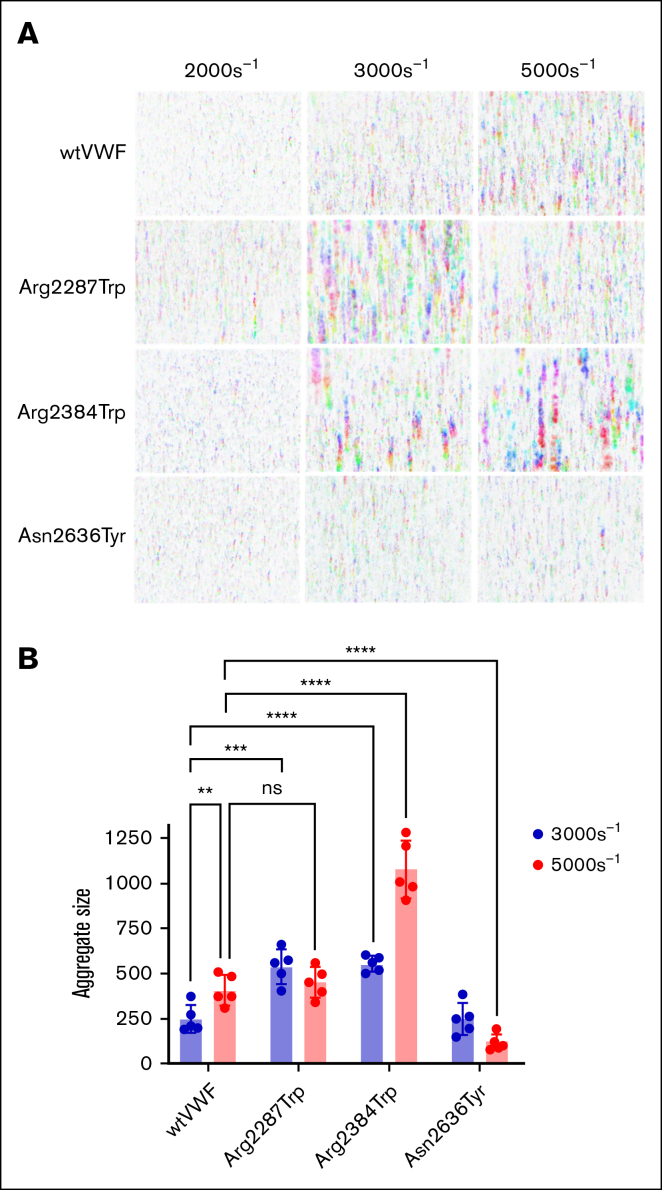
**Formation of rolling VWF-platelet aggregates.** (A) Ibidi VI^0.1^ flow slides coated with 30 μg/mL recombinant VWF were perfused with plasma-free blood supplemented with 5 μg/mL VWF (either wild type or variants as stated) for 60 seconds at 1000 per second, 60 seconds at 2000 per second, 60 seconds at 3000 per second, 60 seconds at 5000 per second. The final 15 seconds of each shear rate were recorded at ∼21 frames per second. A total of 100 frames of recording were processed using ImageJ to remove stationary background objects and track only dynamic objects over the 100-frame recording. Representative images from 5 separate experiments are shown. (B) VWF-platelet aggregate size was determined using ImageJ at 3000 per second and 5000 per second. Error bars represent the mean SD of 5 biological repeats. ∗∗*P* < .001; ∗∗∗*P* < .0001; ∗∗∗∗*P* < .00001. ns, not significant.

To further analyze the impact of the C-domain variants, we performed in vitro flow assays to investigate VWF-mediated platelet capture to collagen under a high shear rate (1500 per second) and pathological shear rate (5000 per second). At 1500 per second, all the variants behaved similarly to wtVWF, promoting a comparable amount of platelet capture to collagen (Figure 3A-B). Interestingly, at 5000 per second the p.Arg2287Trp variant promoted enhanced platelet capture, whereas reduced platelet capture was observed with the p.Asn2636Tyr, p.Thr2647Met, and p.Gly2705Arg variants (Figure 3C-E). Virtually no platelet capture was observed at either shear rate in the absence of VWF (supplemental Figure 3). Previously, the GoF activity of p.Phe2561Tyr was identified by its ability to form rolling platelet-VWF aggregates, which is a good experimental indication of the response of VWF to shear forces.^30^ In this assay, plasma-free blood (and thus lacking ADAMTS13) is supplemented with VWF and perfused over a VWF-coated surface at increasing shear rates. Typically, rolling VWF aggregates begin to emerge at ∼3000 per second and are normally prominent by 5000 per second^30^ (Figure 4A) Strikingly, the p.Arg2287Trp variant formed extremely large aggregates at 3000 per second that were significantly larger than aggregates formed with wtVWF at the same shear rate (Figure 4A-B). At 5000 per second aggregates formed with Arg2287Trp that were highly embolic. Furthermore, the p.Arg2384Trp variant formed significantly larger aggregates at 3000 and 5000 per second when compared to those formed by wtVWF (Figure 4A-B). In contrast, the p.Asn2636Tyr variant formed fewer rolling aggregates (Figure 4A), and these were also significantly smaller at 5000 per second (Figure 4B). Except for Phe2561Tyr, all of the other variants formed rolling VWF-platelet aggregates similar to wtVWF. In keeping with previous reports, the Phe2561Tyr variant also demonstrated GoF-like activity (supplemental Figure 4). Together, these data demonstrate that the p.Arg2287Trp and p.Arg2384Trp variants may confer GoF-like activity to VWF.

### Analysis of dimeric VWF unfolding using optical tweezers

**Figure 5.**
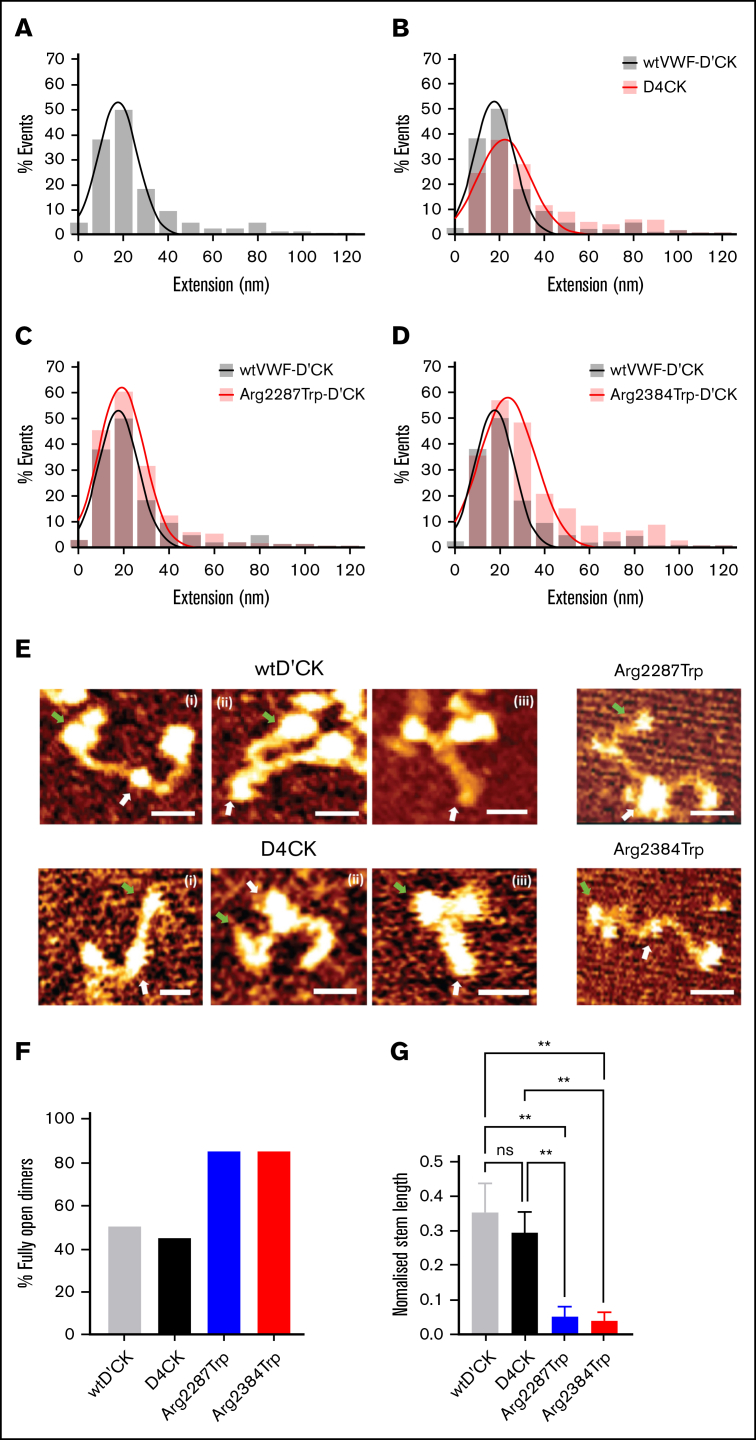
**Optical tweezer pulling and atomic force imaging of the VWF stem.** (A-D) N-terminal Avi (biotinylation) and Spy-tagged hetero-D′CK dimers were expressed in HEK293T cells and purified using nickel affinity chromatography. Proteins were captured on a fixed bead and a laser-trapped bead under an optical tweezer microscope. The laser-trapped bead was allowed to approach and retract from the fixed bead at various pulling speeds from 50 to 500 nM/s. For each protein, >400 positive traces were obtained at each pulling speed. A trace was considered positive if it demonstrated at least 1 unfolding event. All extension events over the 5 pulling speeds were pooled, and the frequency distribution of extensions was determined using Prism using 10nM bins. The number of events was normalized against the number of pulling traces analyzed to account for multiple unfolding events in a single trace and was plotted as frequency distribution histograms to a Gaussian fit. (E) wtVWF-D′CK and D4CK dimers were imaged using AFM. Three example AFM scans are shown for wtD′CK and VWF-D4CK; (i) fully open stem, (ii) partially open stem, and (iii) fully closed stem. Example stem images shown for Arg2287Trp and Arg2384Trp that were predominantly in the open state. White arrows highlight the C-terminal domain of the VWF stem formed via the CK domain. Green arrows represent the N-terminal domains from D4 through to the D′ domain. Scale bar, 20 nm. (F) Frequency of fully open dimers for wtVWF-D′CK, VWF-D4CK, Arg2287Trp, and Arg2384Trp. (G) Stem length was normalized to the distance between the CK domain and the beginning of higher N-terminal domains of the 2 monomers. A normalized stem length of ≥1 indicated a fully closed stem, while a normalized stem length of 0 indicated a fully open stem. ∗∗*P* < .01. ns, not significant.

To explore potential mechanisms behind the GoF-like activity of the p.Arg2287Trp and p.Arg2384Trp variants, we conducted optical tweezer pulling experiments on D′CK dimers to examine the unfolding dynamics of the VWF stem structure (supplemental Figure 1). wtVWF-D′CK displayed a broad range of unfolding extensions between 10nM and 50nM with a weak shoulder at 70 to 80 nM (Figure 5A). The VWF-D4CK protein, which isolates the D4-CK region, showed a much clearer second peak in this range, and both constructs exhibited more 70nM to 100nM events, consistent with the reported ∼80nM separation of the D4 assembly. Although it was expected that VWF-D4-CK would show a single unfolding event ∼80nM corresponding to D4-D4 dissociation, both wtVWF-D′CK and VWF-D4-CK primarily exhibited short extensions (<35nM), along with additional events ranging from 30nM to 90nM (Figure 5B). These shorter events likely result from unfolding within the C-domains or rearrangements inside the D4 assembly. Next, we investigated the impact of the p.Arg2287Trp and p.Arg2384Trp mutants. Interestingly, although the p.Arg2287Trp variant demonstrated a broadly similar distribution of unfolding events similar to wtVWF-D′CK, it also exhibited more unfolding extension events over the shorter-range lengths but had fewer 80nM events, suggesting decreased D4-D4 interactions (Figure 5C). In contrast, the p.Arg2384Trp variant showed more unfolding events >20nM compared to wtVWF-D′CK and had an increase in events over 80nM, indicating more D4-D4 uncoupling events (Figure 5D). Overall, these data suggest that both variants have altered unfolding properties that could contribute to their GoF-like activity.

### AFM imaging

Based on the data from the OT experiments, we hypothesized that both variants would have an altered stem conformation and used AFM to directly visualize the protein conformation. In keeping with previous reports, 3 main stem states were observed: the stem partially closed, the stem fully closed, or the stem fully open (Figure 5E). Based on these states, the normalized stem length could be determined, with a value of 1 indicating the stem was fully closed, and a value of 0 indicating the stem was fully open. For wtVWF-D′CK and VWF-D4CK, ∼50% of the dimers were found to be in a fully opened stem state (Figure 5F). Significantly, both p.Arg2287Trp and p.Arg2384Trp variants favored the open stem conformation (Figure 5E-F), with ∼85% of the molecules exhibiting a fully open stem (Figure 5F). Finally, analysis of the average normalized stem length showed that both p.Arg2287Trp and p.Arg2384Trp variants demonstrated a significant difference in stem state, with the open conformation favored (Figure 5G). Combined with the OT data, this demonstrates that both variants favor an open stem conformation that leads to enhanced activity under shear stress.

### Impact of p.Arg228Trp and p.Arg2384Trp on the VWF stem structure

**Figure 6.**
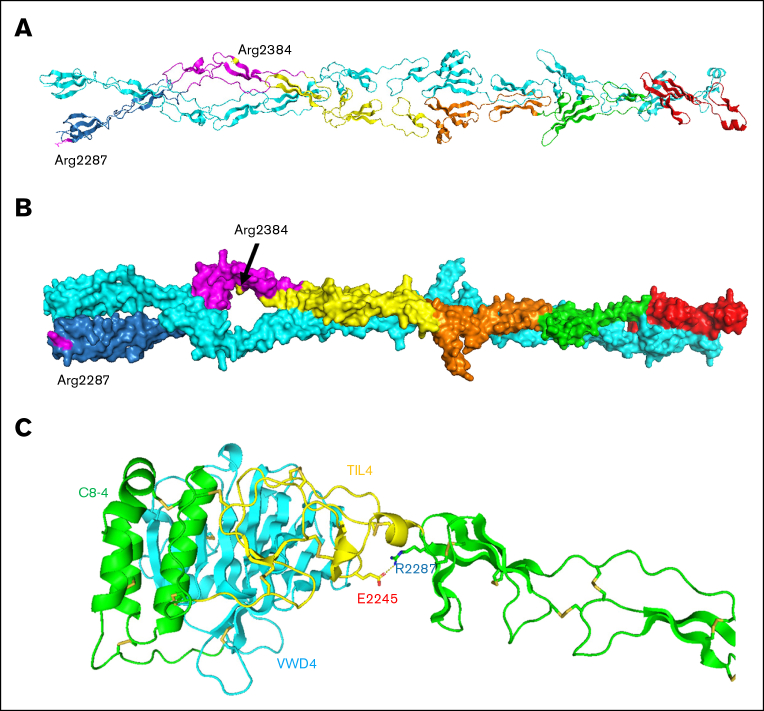
**Predicted model of the VWF stem structure.** Alphafold-3 was used to model the C1-C6 domains. (A) Ribbon representation of C1-C6 and (B) surface representation of the C1-C6 stem. C1, dark blue; C2, magenta; C3, yellow; C4, orange; C5, green; C6, red. The relative position of Arg2287Trp and Arg2384Trp is shown. (C) Alphafold-3 model of the D4-assembly and C1 domain showing Arg2287 and its putative interaction with Glu2245 in the TIL-4 sub-domain of the D4 assembly.

Currently, no structural data exists on the C1 and C2 domains of VWF or indeed the entire stem region. Therefore, we prepared a model of the C1C6 dimer using Alphafold-3. Interestingly, the addition of the CK or D4 assembly in Alphafold-3 did not yield a suitable dimeric stem model. Based on our C1C6 model, Arg2287 occurs at the top of the C1 domain, and Arg2384 is located on the outer face of the C2 domain; neither residue could be predicted to aid dimer formation (Figure 6A-B). Although the impact of Arg2384Trp could not be predicted, we further generated a model of the D4-C1 monomer to explore Arg2287Trp (Figure 6C). Interestingly, in this model, Arg2287 forms an interaction with E2245 in the TIL-4 region of the D4 assembly (Figure 6C). Potentially, the loss of this interaction could make the C1 domain more flexible relative to the D4 assembly, affecting formation of the D4-D4 interaction.

## Discussion

Previously, 3 C-domain variants have been reported with GoF-like activity: p.Phe2561Y, p.Pro2555Arg and p.Gly2705Arg.^30^, ^31^, ^32^ In the present study, we have identified 2 further variants, p.Arg2278Trp and p.Arg2384Trp, located in the C1 and C2 domain respectively, that also confer GoF-like activity to VWF. Additionally, we have also observed 2 further variants that exhibit apparent reduced function, but only under pathological shear stress conditions. While p.Arg2278Trp and p.Arg2384Trp are low-frequency variants, the allele frequency of both is more prevalent in the African-American population (0.8% and 0.2%, respectively). Both are reported to be of unknown clinical significance. A previous study had concluded p.Arg2287Trp was probably to cause VWD based on reduced in vitro expression.^41^ While we also observed reduced expression, the index case reported plasma VWF was measured at 49 IU/dL, which would now fall into the low VWF classification.^42^ Moreover, the precise bleeding history of patients associated with this mutation is not reported. Interestingly, we also observed reduced expression for p.Arg2313His, p.Arg2384Trp and p.Tyr2666Met, indicating they may cause reduced VWF levels.

Except for p.Arg2287Trp, all the variants behaved normally under static conditions. Slightly reduced binding to GPIIbIIIa was seen with p.Arg2287Trp, although statistically significant, binding isotherms were similar to wtVWF, and it was only the end point analysis that demonstrated a difference in binding; it is therefore unlikely to have any effects of note on VWF function. Interestingly, p.Arg2287Trp bound to GoF-GPIbα significantly more than wtVWF. The use of GoF-GPIbα eliminated the need for ristocetin and unfolding of the A1 domain, and we could therefore attribute any differences to the C-domain mutation. The enhanced binding to GPIb could potentially contribute to its GoF-like activity and may suggest that the C-domains have a role in modulating GPIb binding. In flow assays probing VWF-mediated platelet capture to collagen, no differences were seen at 1500 per second, representing a high, but physiological shear rate. However, at the pathological 5000 per second, p.Arg2287Trp mediated increased platelet capture whereas p.Asn2636Tyr, p.Thr2647Met, and p.Gly2705Arg all had reduced capture. The formation of rolling VWF-platelet aggregates acts as a useful assessment of VWF responsiveness to shear forces. Subsequently, p.Arg2287Trp and p.Arg2384Trp mediated enhanced VWF-platelet aggregate formation at lower shear thresholds, indicating GoF activity. Conversely, p.Thr2647Met and p.Asn2636Tyr had reduced function in this assay, suggesting that, in contrast to GoF function variants, some VWF mutations may invoke loss of high shear function. The mechanism and significance of this will be the subject of follow-up studies. The flow-based data also highlighted assay-specific differences; for example, over collagen at 5000 per second p.Arg2287Trp showed increased function, but p.Arg2384Trp and the previously reported p.Phe2561Tyr variants did not; however, all 3 variants were more responsive to shear stress in the VWF-platelet rolling aggregate assay.

Why GoF-like effects are not readily seen over collagen surfaces is not apparent. However, it may reflect the extent to which VWF undergoes a conformational change. It is noteworthy that the loss of function variants had a reduction in VWF-mediated platelet capture at 5000 per second (p.Thr2647Met and p.Asn2636Tyr), indicating that the C-terminal domains are in some way involved in collagen binding and platelet capture at high shear. For VWF-platelet rolling aggregate formation, the initial precipitating events are platelet capture to immobilized VWF, followed by soluble VWF interacting with the immobilized platelet and further platelet and VWF recruitment.^43^ In the case of the GoF mutants, the shear threshold is lowered for soluble VWF to unfurl and subsequently interact with platelets. The result of VWF being “open” is longer soluble strings that can subsequently capture more platelets and form rolling aggregates. This open molecule may also be more prone to self-association, further enhancing the mass of VWF to capture platelets, although this conjecture requires further investigation. Conversely, when perfused over collagen, although the GoF mutants may be more responsive to shear forces, the effect in some cases may be limited by the molecule binding to collagen via multiple A3 domains. Further work to establish if the GoF mutants can interact more readily with immobilized collagen under flow conditions would shed light on this. It should be stated that our observations of the p.Gly2705Arg variant are conflicting with Chen et al, since we did not observe any GoF activity, and indeed, we observed less function over collagen at pathological shear. However, it should be noted that in their study, the variant was analyzed under flow conditions at 1800 per second using plate and cone aggregometry, different to our experiments.^31^ Interestingly, p.Gly2705Arg has been shown to be protective against deep vein thrombosis, which would potentially conflict with any GoF activity.^44^

To determine a mechanism for the GoF activity, OT and AFM were performed, highlighting several important findings. Like previous observations using AFM, we observed a broad Gaussian distribution of the size of unfolding events with a major peak around ∼20nM and a minor peak around ∼80nM.^27^^,^^45^ Since OT offers increased sensitivity over AFM, the 20nM to 30nM events most likely represent A1 and A2 domain unfolding events,^38^^,^^46^ whereas the ∼80nM event is the dissociation of the previously reported D4-D4 interaction.^27^ Furthermore, the analysis of the VWF-D4-CK protein (thus lacking A1 and A2 domains) showed that novel unfolding events also occur within this region. This agrees with previous studies where low forces were predicted to break the interactions between C-domains.^28^ Although no biochemical data have demonstrated a direct interaction between VWF C1C6 stems, cryoelectron microscopy and AFM images imply that this interaction occurs.^24^, ^25^, ^26^, ^27^, ^28^^,^^47^ Thus, the smaller unfolding events we observed with the VWF-D4CK protein likely represent an opening or “unzipping” of the C1-C6 stem. In addition to this, they may also represent extensions of the C-domains themselves and unfolding of the D4 assembly. To our knowledge, this is the first report of a VWF molecule comprised of the C-terminal domains being analyzed by OT. Interestingly, both variants had an increased number of unfolding events compared to wtVWF. This is likely attributed to an increased number of extension/unfolding events in the D4-C6 domains, although we cannot rule out increased A1 and/or A2 extensions. The lack of the 80nM event with p.Arg2287Trp also suggests that the D4-D4 interaction is lost with this variant, while the increased events at ≥80nM with p.Arg2384Trp indicate that the D4-D4 interaction is easily disrupted. Together, this would suggest that for both variants, the stem is more readily opened, resulting in enhanced function. AFM imaging supports this notion, showing that both molecules favor the open stem conformation. Why these mutants affect VWF in this way is not entirely clear. The reduction in expression of both variants may suggest that alterations in protein folding alter stem formation or stability. While only speculative, the Alphafold-3 model of the D4C1C6 monomer suggests that p.Arg2287Trp may mediate interaction of the C1 domain with the D4 assembly; this could therefore destabilize the D4-D4 interaction, which is supported by the OT observations. p.Arg2384Trp is predicted to be surface exposed; therefore, it is likely that the loss of charge affects dimer-dimer interactions within the stem. Although the p.Arg2287Trp and p.Arg2384Trp mutations are much less common than the p.Phe2561Tyr mutation, it will be interesting to explore if there is any putative link with cardiovascular disease. However, aside from any clinical significance, our data demonstrate that, like the C4 and C6 domains, the C1 and C2 domains can harbor mutations that promote enhanced function by modulating stem function and thus response to shear forces. Moreover, we show, to our knowledge, for the first time that mutations in the VWF protein can result in a molecule with reduced function, but only under pathological conditions, at least in vitro. In conclusion, our data shows that the C-domains of VWF play a critical role in VWF function that only becomes apparent under flow conditions.

Conflict-of-interest disclosure: The authors declare no competing financial interests.

## Supplementary Material

Supplemental Methods, Figures, and References
