# Supplementary material for: Identification of missense variants in the C-domains of von Willebrand factor that cause gain-of-function–like activity
Source: Blood Adv. 2026 Mar 6;10(12):4334–46. doi: 10.1182/bloodadvances.2025018101 (PMC13276585; doi:10.1182/bloodadvances.2025018101)
Supplement: Supplemental Methods, Figures, and References [file BLOODA_ADV-2025-018101-mmc1.pdf]

Supplementary Methods

Expression of VWF

We have previously generated and described the expression vector pcDNA3.1-FL-VWF that encodes for full length VWF.[1] Mutations were introduced into this vector using the QuickChange Site directed mutagenesis kit (Agilent) as per manufacturer instructions, and as previously described.[2] To assess VWF expression, HEK293T cells were seeded into 6 well tissue plates at 6 x 10^5 cells/well, 24 hours before transfection. Cells were transfected with 1 µg/ml plasmid DNA in a total volume of 2ml using polyethylenimine as the transfection reagent. Media and cell lysate samples were harvested 72 hours post transfection. For expression studies, 5 separate transfections were performed in duplicate. In some experiments cells were co-transfected with the vector pEGFP encoding for green fluorescent protein (GFP) to monitor transfection efficiency. GFP expression in cell lysates was determined by SDS-PAGE and subsequent western blotting and probing with anti-GFP-HRP antibodies. For large scale VWF expression, 10 – 15 confluent T175 flasks per variant were transfected and media collected 72 hours post transfection. Media was concentrated ~100-fold using 100kDa cut-off centrifuge spin column concentrators.

Binding to gain-of-function Glycoprotein Ibα (GoF-GPIbα)

The vector ET8-GPIba-high-6His that encodes for the first 290 amino acids of glycoprotein Ibα containing the Gly233Val and Met239Val mutations that promote binding to VWF without the need for ristocetin, was purchased from Addgene.[3] Protein was expressed in HEK293T cells and purified using nickel affinity chromatography. To measure VWF binding, 96-well MaxiSorp™ plates were coated with 2.5µg/ml of an anti-human CD42b antibody (Biolegend, UK) diluted in phosphate buffered saline (PBS) overnight at 4°C. Following washing three times with PBS supplemented with 0.1% Tween-20 (PBS-T), wells were blocked with 2% bovine serum albumin in PBS for 60 mins at room temperature. Wells were then washed again and incubated with 5 µg/ml GoF-GPIbα for 2 hours at room temperature. VWF samples were prepared using a 7-point serial dilution from 5µg/ml (~18.5nM) in PBS. After washing off unbound GoF-GPIbα, VWF was added to the wells in duplicate and incubated overnight at 4°C. Subsequently the wells were washed three times with PBS-Tween and bound VWF detected with polyclonal anti-VWF-HRP antibodies (DAKO, UK) diluted to 1 µg/ml in PBS-T for 1 hour at room temperature. After a final three washes 150 µl of SigmaFast OPD substrate (Sigma, UK) was added to each well. The reaction was stopped with 50µl of 2M H_2SO_4 (Sigma, UK) Absorbance was recorded at 492nm using a spectrophotometer (UV-1601 spectrophotometer, Shimadzu).

Binding to purified glycoprotein IIbIIIa (GPIIbIIIa)

GPIIbIIIa purified from platelets was purchased from Enzyme Research Labs (UK). 96-well MaxiSorp™ plates were coated with 5µg/ml of GPIIbIIIa diluted in PBS and left to incubate overnight at 4°C. Samples were prepared using a 7-point serial dilution with a starting concentration of 5µg/ml (18.5nM) in assay buffer (20mM Tris, 100mM NaCl, 1mM $CaCl_2$, 1mM $MgCl_2$). The same buffer was used in duplicate wells as a blank control. The samples were left to incubate overnight at 4°C. All washing, blocking and detection steps were performed similarly to the GoF-GPIbα assay.

Generation of N-terminal tagged dimeric VWF expression vectors

The vectors pcDNA3.1-Avi-VWF-D'CK-His and pcDNA3.1-Spy-VWF-D'CK-His encoding for the D'CK domains with an N-terminal Avi or Spy tag and a C-terminal His tag were generated using the HiFi assembly kit (NEB, UK). Overlapping primers were designed using the NEBuilder tool to amplify the VWF signal peptide coding sequence (encoding for amino acids 1 to 22) with a 5' EcoRI restriction site, the Avi or Spy tag sequence and the D'-A2 coding sequence, encoding for amino acids 764 to 1600 which is part way through the A2 domain. A naturally occurring KpnI restriction site is located around the sequence encoding amino acid 1584 and can used for cloning. PCR reactions to amplify these fragments were performed using NEB Q5 polymerase. Following PCR and DNA gel electrophoresis, bands were excised from the gel and extracted using the NEB gel extraction kit and assembled in the vector pGEM 7+ that had been linearized with XhoI and KpnI restriction enzymes. Following transformation and subsequent bacterial culture and mini-prep DNA purification, the resulting vectors (pGEM-VWF-SP-Avi-D'A2 and pGEM-VWF-SP-Spy-D'A2) were verified by DNA sequencing (Whole plasmid sequencing, Genewiz, UK).

Previously we generated the expression vector pcDNA3.1-FL-VWF which encodes for full length VWF in a modified pcDNA3.1 vector.[1] This vector contained a 5' EcoRI site and a stop codon immediately after the last VWF codon followed by a 6x His tag sequence, a stop codon and an Age I restriction site. Site directed mutagenesis was used to deleted the stop codon from this vector (pcDNA3.1-FL-$VWF^{no\text{-}stop}$) allowing in frame expression of the His-Tag. Subsequently, pGEM-VWF-SP-Avi-D'A2 and pGEM-VWF-SP-Spy-D'A2 were digested with EcoRI and KpnI to remove the SP-Avi or Spy-D'A2 fragment and this was ligated into pcDNA3.1-FL-$VWF^{no\text{-}stop}$ digested with the same enzymes. The resulting pcDNA3.1-Avi-VWF-D'CK-His and pcDNA3.1-Spy-VWF-D'CK-His vectors were verified by whole plasmid sequencing. Vectors encoding for Avi or Spy-VWF-D4-CK-His to generate VWF molecules spanning the D4-CK domains with N-terminal Avi or Spy tags and C-terminal His-tags were generated in pcDNA3.1 using the GeneArt service (Invitrogen, UK).

Expression and purification of tagged VWF dimers

Dimeric VWF was expressed as hetero-bifunctional dimers by transfecting HEK293T cells with pcDNA3.1-Avi-VWF-D'CK-His and pcDNA3.1-Spy-VWF-D'CK-His (or pcDNA3.1-Avi-VWF-D4CK-His and pcDNA3.1-Spy-VWF-D4CK-His) and pcDNA-BirA in a ratio of 1:1:0.1 in Opti-MEM supplemented with 100µM D-biotin filtered biotin. The co-expression with BirA allowed for biotinlyation of the Avi-tag. Four days post transfection condition media was harvested and VWF dimers purified by Nickel Affinity chromatography. SDS-PAGE under reducing and non-reducing conditions and Coomassie staining was used to confirm purity and dimerization. Pure eluted fractions were pooled and concentrated 20-fold using Amicon 100kDa centrifugal concentrators and buffered exchanged into phosphate buffered saline. Biotinylation was confirmed by capturing the VWF dimers to an anti-VWF coated 96 well plate and detecting with streptavidin-HRP antibodies. Based on the dimerization process it was assumed that 1/3 of the expressed dimers would be heterogeneous.

Optical Tweezer analysis

Preparation of Streptavidin Beads

For single-molecule OT experiments, micron-sized (2-5µm) polystyrene beads were used to exert forces on the protein of interest. Briefly, 2.0µm carboxyl-polystyrene beads (Spherotech, USA) were firstly, covalently coupled with 100mg/ml streptavidin (Invitrogen, USA) using the PolyLink coupling kit (Polysciences, USA) following the manufacturer's protocol. The streptavidin beads were then incubated with ~1µg/ml of the dimeric VWF proteins for 10 minutes at room temperature and then placed on ice. Simultaneously, a separate batch of streptavidin beads were incubated 1:1 (v/v) with Spycatcher protein previously coupled to a biotin-DNA handle via thiol group attachment.

OT Experimental Set-up

The streptavidin beads coated with dimer VWF were injected into the upper chamber channel while the SpyCatcher, coupled to a biotin DNA handle, was injected into the lower channels using a 1ml syringe. The liquid environment in the middle channel was buffered with TBS (10mM Tris, 150mM NaCl, pH 7.5) at room temperature. A single streptavidin bead containing the SpyCatcher DNA handle was then trapped using the dual-laser trap and brought to the centre of the middle channel. The streptavidin bead coated with VWF sample protein was then fixed to the micropipette. Through this interaction, the VWF dimer was pulled by applying force (0-100pN) through an approach and retract mechanism at various pulling speeds (50, 100, 200, 400, 500nm/s). The stretching force increases linearly with the pulling distance. The force and bead-bead distances were detected by the position-

sensitive photodetector, and force-extension curves were generated. The force-extension curves and frequency of the unfolding events were analysed using MATLAB.

Supplementary Figures

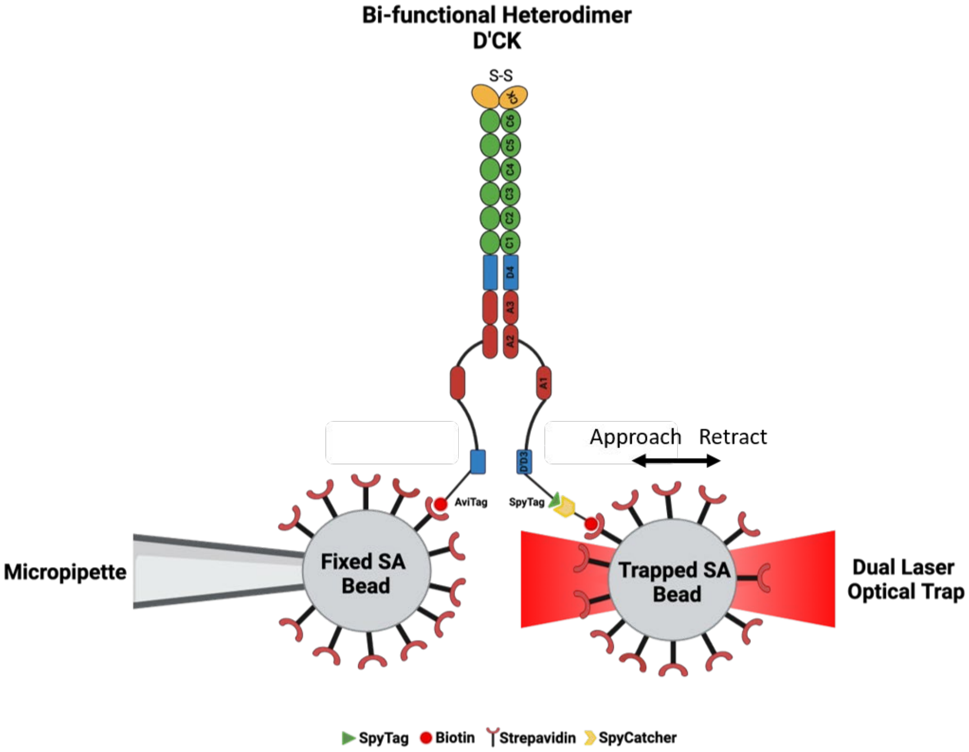

Supplementary Figure 1. Single-molecule optical tweezer set up. Carboxyl-polystyrene beads coated with Streptavidin *(SA beads)* and the desired VWF variant were fixed onto a micropipette by suction. A second streptavidin bead coated with the Spycatcher DNA handle was trapped and positioned using the counter-propagating dual-laser trap. The VWF dimer was pulled at various speeds, 50, 100, 200, 400, and 500 nm/s.

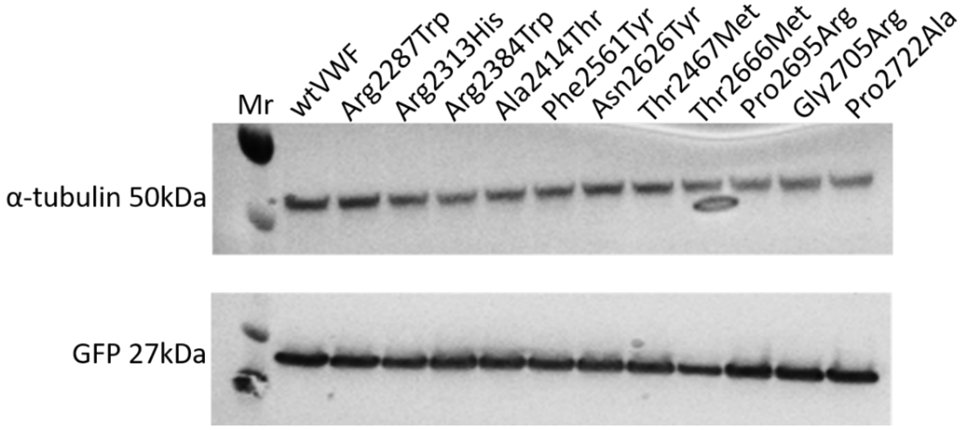

Supplementary Figure 2. Co-expression of GFP with VWF. HEK293T cells were co-transfected with expression vectors for wtVWF or its variants and pEGFP encoding for GFP. Cell lsyates were harvested after 72 hours transfection and subjected to SDS-PAGE, followed by western blotting for GFP and α-tubulin as a house keeping protein. Despite differences in VWF expression in cell lysate and media samples as analysed by ELISA, GFP expression was uniform across the transfections.

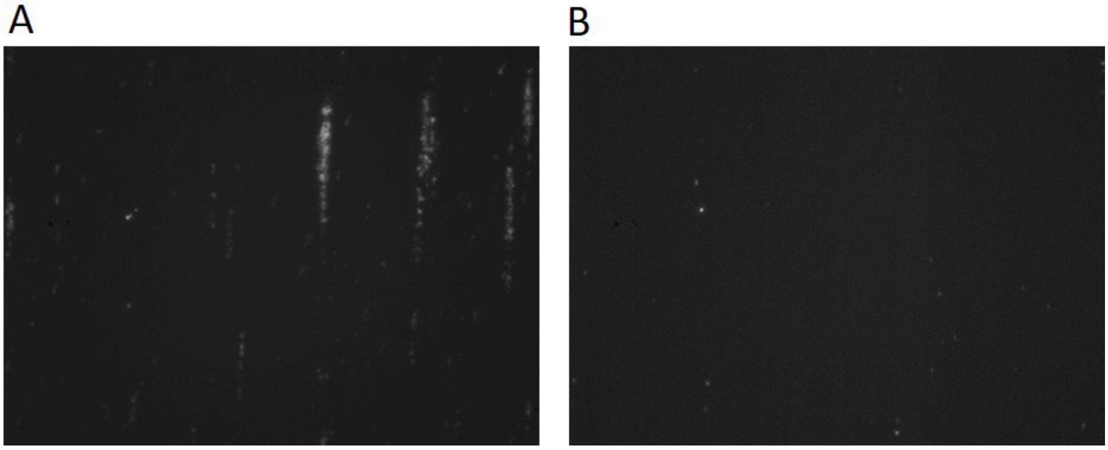

Supplementary Figure 3. VWF mediated platelet capture to collagen controls. Washed red blood cells and platelets without the addition of VWF were perfused over type III collagen surfaces at (A) 1500s^{-1} and (B) 5000s^{-1}. Images were captured after 5mins of perfusion. At 1500s^{-1} minimal platelet capture was observed and virtually no visible platelet capture was seen at 5000s^{-1}.

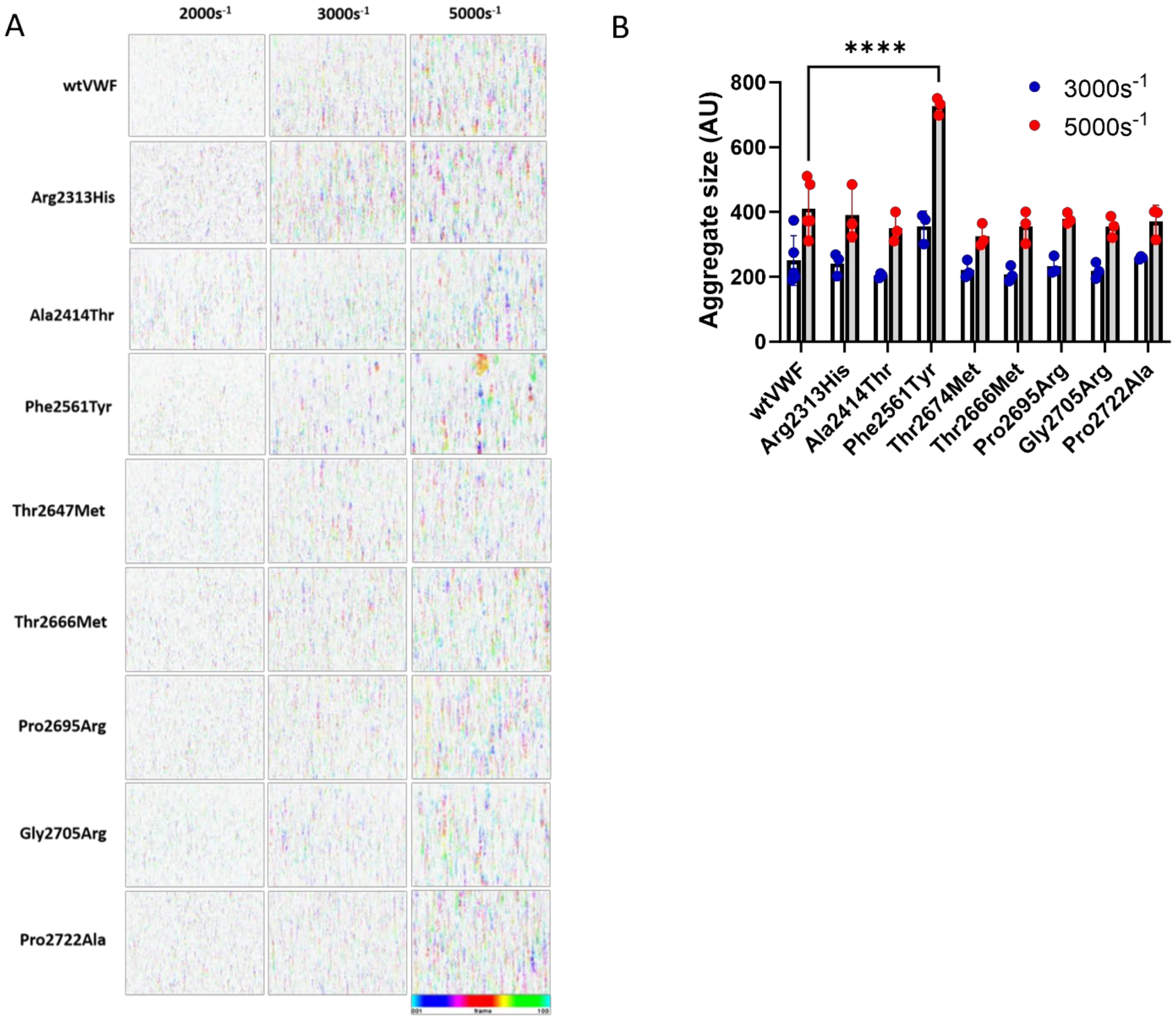

Supplementary Figure 4. Formation of rolling platelet-VWF aggregates. (A) Ibidi VI$^{0.1}$ flow slides coated with 30 µg/ml recombinant VWF were perfused with plasma-free blood supplemented with 5 µg/ml VWF (either wild type or variants as stated) for 60 secs at 1000s^{-1}, 60 secs at 2000s^{-1}, 60 secs at 3000s^{-1}, 60 secs at 5000s^{-1}. The final 15 secs of each shear rate were recorded at ~21 frames per second. 100 frames of recording were processed using ImageJ to remove stationary background objects and track only dynamic objects over the 100-frame recording. Representative images from 5 separate experiments are shown. (B) VWF-platelet aggregate size was determined using ImageJ at 3000s^{-1} and 5000s^{-1}. (Error bars represent the mean SD of 3-4 biological repeats **** p=<0.00001). With the exception of the previous described Phe2561Tyr variant, the remaining variants had no impact on the formation of rolling VWF-platelet aggregates.

Supplementary References

1. McKinnon TA, Goode EC, Birdsey GM, et al. Specific N-linked glycosylation sites modulate synthesis and secretion of von Willebrand factor. *Blood*. 2010;116(4):640-648.
2. Nowak AA, Canis K, Riddell A, Laffan MA, McKinnon TA. O-linked glycosylation of von Willebrand factor modulates the interaction with platelet receptor glycoprotein Ib under static and shear stress conditions. *Blood*. 2012;120(1):214-222.
3. Jiang Y, Fu H, Springer TA, Wong WP. Electrostatic Steering Enables Flow-Activated Von Willebrand Factor to Bind Platelet Glycoprotein, Revealed by Single-Molecule Stretching and Imaging. *J Mol Biol*. 2019;431(7):1380-1396.
